# Design and development of a combined seedbed compactor and teff seed cum fertilizer drill machine

**DOI:** 10.1016/j.heliyon.2024.e36856

**Published:** 2024-08-24

**Authors:** Mulu Marie Takele, Gedion Zelalem Dires, Smegnew Moges Mintesnot, Geta Kidanemariam Wagaw

**Affiliations:** a Agricultural Mechanization Staff of Bahir Dar Institute of Technology, Bahir Dar, Ethiopia; b Researcher and Workshop Manager at Gedefaw Yismaw Automotive and Trailer Manufacturing, Bahir Dar, Ethiopia

**Keywords:** Broadcasting, Compactor, Drill, Teff, Teff seed-cum-fertilizer and trampling

## Abstract

Teff, which is surged in other continent and a cornerstone Ethiopian grain used as injera, suffers from low yield due to outdated sowing practices and minimal use of modern fertilizers. The traditional method is inefficient, squandering seeds and fertilizer, and relying on a large number of animals for trampling. Researchers addressed this challenge by creating a new machine that combines a seedbed compactor with a tool that sows both teff seeds and fertilizer. They conducted lab tests to analyze how different settings affected the machine's performance. These settings included the speed (varying from 2.11 to 3.14 km per hour), the amount of fertilizer and seeds in the hopper (full, half, or quarter full), and the shape of the opening in the metering plate (circular, square, or a specific angled square). The weight of the compactor was also adjusted (40, 50, or 60 kg).

Analysis of the results using Design Expert-13 software showed that a circular opening in the metering plate combined with a speed of 2.6 km per hour yielded optimal results, distributing 4.2 kg of seeds and 96.2 kg of fertilizer per hectare. Additionally, the compactor filled with 50 kg of sand generated a compaction force of 483.7 N. These findings satisfy the design requirements of applying 3–5 kg of seeds, 50–100 kg of fertilizer, and achieving a compaction force of 480 N per hectare. This method significantly reduces seed waste by 81 % compared to traditional techniques.

## 1 Introduction

Teff's popularity has surged in recent years. This is likely due to its perceived health benefits, leading to its cultivation in new regions like North America, China, India, Australia, and the UK. Additionally, teff remains economically valuable in Ethiopia, fetching a higher price compared to other grains. In other countries, the growing desire for healthier options drives consumers to pay a premium for teff-based products [1]. Ethiopia is a teff powerhouse. A significant portion of its farmland (32 %, or 3 million hectares) is dedicated to teff cultivation, resulting in a substantial grain production (5.2 million tons, representing 21 % of the country's total) [2]. Notably, teff reigns supreme among Ethiopian cereal crops, being the primary source of income for farmers due to its high market demand and prices. This is particularly evident in North East Amhara, where the study was conducted, with teff ranking first and second in both area coverage and production volume [3], respectively. However, despite its dominance, teff productivity in Ethiopia faces challenges. These include a lack of high-yielding varieties, limited use of modern agronomic practices, and reliance on traditional methods. Additionally, declining soil fertility and unpredictable rainfall patterns contribute to lower yields. Fortunately, solutions exist. Implementing a combination of improved teff varieties, optimized tillage techniques, row sowing methods, proper fertilizer and seed application rates, and effective weed control can significantly boost teff yields without incurring excessive production costs. Currently, farmers often resort to broadcasting seeds at high densities (>25 kg/ha) to improve seed establishment, but this approach is not the most efficient [4,5]. Unfortunately, the common practice of high-density broadcasting seeds comes with drawbacks. It increases seed costs due to excessive use. Additionally, by crowding plants together, it creates competition for resources like nutrients and sunlight, ultimately hindering their growth and reducing overall yield. This method also proves inefficient in terms of resource utilization [6]. Traditional teff cultivation relies on multiple tillage passes (3–5 times) with oxen for plowing and compaction. This dependence on animal power not only increases the cost of broadcast sowing but also proves cumbersome. In contrast, row sowing offers several advantages. It promotes efficient resource use by reducing seed waste and facilitating easier farm activities like weeding and fertilizing. Additionally, row sowing allows for better control over seed depth and placement, ultimately leading to improved germination and crop establishment [12] [7]. Even though row sowing improves teff productivity, adoption rates are still low because it requires more labor. Teff seeds, with a thousand grain weight of approximately 0.265 g, are small and difficult to spread evenly. Consequently, 25–30 kg/ha of seeds are advised for broadcasting. However, it is customary to disperse teff at a rate of 40–50 kg/ha. Row planting at a reduced seed rate of 2.5–3.0 kg/ha minimizes plant competition and enables optimal management, including weeding [8]. Farmers' experience has shown that trampling the teff field requires more animal power. The field during peak seeding time to observe severe trampling, and the results show that the power required for trampling a quarter of a hectare of teff requires 15 to 20 animals for 6 h. It is used by farmers to promote germination and establishment, firm up the seed bed, keep the soil surface from drying out, and rid the seed bed of weeds [9]. There are different technologies developed for the production of teff, especially for its sowing and compaction. Teff row planters have developed [8,10] by different researchers. Not only planter but also an animal-drawn compactor has developed [11]. There is a big gap between the action of production and the development of machines in Ethiopia. So, the use of technology is in the infant stage. The combined seedbed compactor and teff seed cum fertilizer drill machine performs its task, such as compacting the seedbed, drilling the seed and fertilizer when the machine is pulled by a pair of animals, and compacting the seed bed to eliminate the need for a large number of animals for trampling, sowing seeds at the required seed rate and depth to minimize seed loss and the effect of the plant lodging, and applying fertilizer at the required amount and manner to minimize environmental effects and fertilizer waste.

## 2 Material and method

### 2.1 Data collection

The necessary data was gathered from various documents, agricultural research reports, observation, literature review, and questions from agricultural experts. Those data were like the agronomical recommended inputs, seed properties, and the specification of what the research needs to achieve.

Through different methods as follows.

• Through observation;
  o Looking at difficulties of Sowing of teff regarding to labor, animal
  o Checking the amount of resources used for broadcasting of teff regarding to loss
  o Is there any technology for sowing of teff which consider the two points
• Investigating what the teff cultivation requires
  o It needs compacted seed bed since it is small seed sized and can't germinate if more soils cover it. So how the farmers compact teff seed bed.
  o Is which method the teff seed is sow and checking the level of importance
• Evaluating the logical concept and literature were evaluated.
  o Referring different sources to know which is important and develop the technology

### 2.2 Development of seed metering mechanism

Vertical seed plates are positioned vertically and have small holes that allow seeds to drop down into the soil. They are typically used for planting small seeds like lettuce or carrots. The metering mechanism helps to meter the seed with its uniform rate and spacing. The physical dimensions of teff and fertilizer is used for designing the size of hole on seed and fertilizer metering device.

Similar to Ref. [12] the tangetial speed of the wheel be:

(1) v=ωxr

where, v is the tangential speed of the wheel (linear speed)

ω is the angular velocity of the metering wheel

r is the wheel radius, assumed to be 17 cm

(2) $$\omega 1=\frac{2\;x\;\pi \;x\;N}{60}$$

where, N is revolution of metering plate.

The number of slot holes on the plate are determined as follows as similar to Ref. [12] $n=\frac{\pi \;x\;D}{i\;x\;x}$ (3)

Where, n is the number of slot holes on metering plate.

D is the diameter of the ground metering wheel

i is the gear ratio (2:1)

x is the slot hole to slot hole spacing.

The diameter of the metering plate (Dr) can be determined from the circumference of circular plate [12].

(4) πDr=nxx

### 2.3 Working principle of the machine

A combined seedbed compactor and teff seed cum fertilizer drill machine (Appendix A, Figure A.3) is pulled by a pair of oxen, and the roller compactor cylinder compresses the teff seedbed. Once the seed bed is opened to the desired depth by the furrow opener, a compactor is utilized to level and compact the soil, resulting in an appropriate seedbed for planting teff seed. To achieve the desired compaction level based on different soil types and degrees of soil moisture, a hollow, empty cylinder is filled with sand, and the weight of the compactor can be adjusted accordingly. While the metering wheel transfers motion to the chain and sprockets, ultimately leading to the rotation of the seed and fertilizer metering shaft. This, in turn, enables the seed and fertilizer metering mechanism to collect the required amount of seed and fertilizer based on predetermined agronomic requirements. The seed and fertilizer are transported through the seed and fertilizer delivery tube to reach the boot.

### 2.4 Experiment setup

**Fig. A.1:**
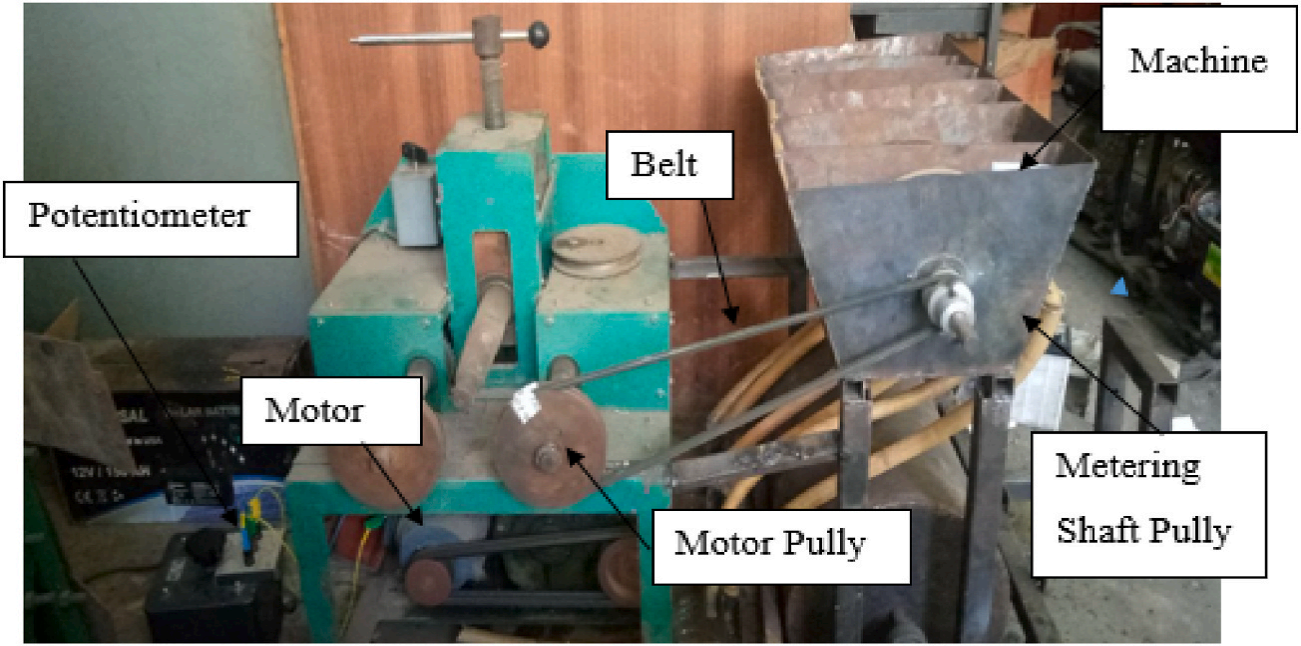
Laboratory setup of testing machine.

**Fig. A.3:**
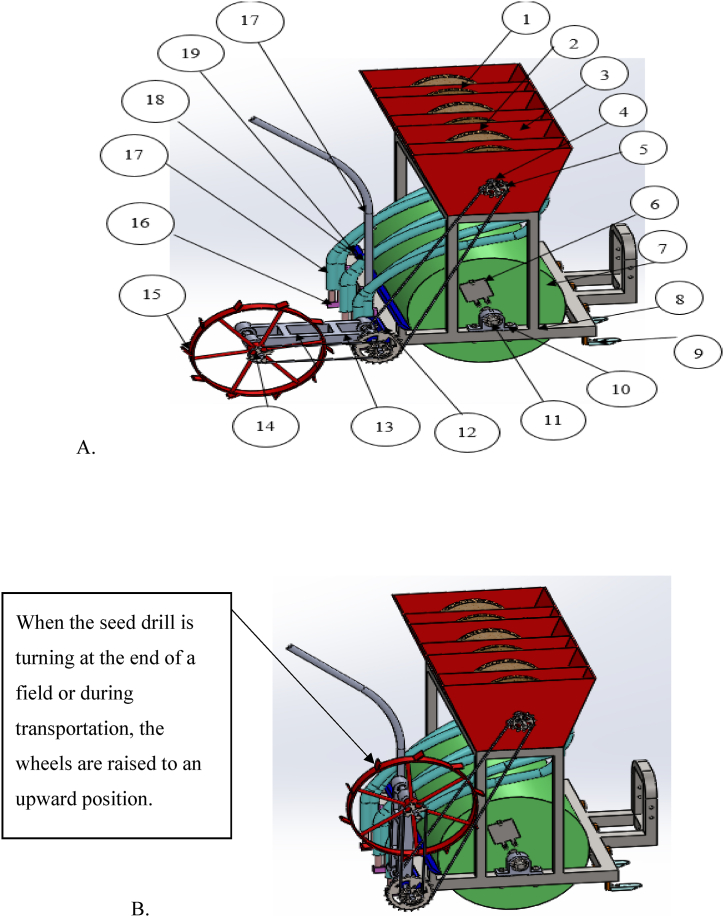
Combined seedbed compactor cum seed and fertilizer drill machine: A. At working and B. upward position at the turning of end of furrow.

The FAO test procedure was used in laboratory tests to calculate the rates of seed and fertilizer in kilograms per hectare (kg/ha) [13]. A prototype machine comprising a motor, pulleys, a belt, a potentiometer, and a metering mechanism made up the experimental setup (Appendix A, Fig. A.1). the shaft which carry metering plate and metering plate were rotated by a motor-driven pulley system. Seed and fertilizer passed through metering plate holes and gathered in a container through centrifugal force. After 3 min for teff seed and 1 min for fertilizer, the amount of seed and fertilizer that had been released was measured. The procedure was timed by a stopwatch, motor speed was regulated by a potentiometer, and pulley revolutions were counted by a tachometer. A balance was used to weigh the collected seed or fertilizer.

### 2.5 Experimental variables

Experiments were performed for three different vertical plates having three different shapes of hole having circular, square, square with 45° inclined in one side of the direction of rotation of metering plate for both seed and fertilizer. The assessment of a vertical plate-type metering mechanism for both seed and fertilizer were performed at three different forward speed of 2.11, 2.5 and 3.14 km/h with three different level of hopper (full, half and quarter) and shape of circular, square, square with 45° inclined.

The combinations of all these independent variables of forward speed, level of hopper and the shape of hole of the metering plate affect the seed and fertilizer rate were taken into consideration. 81 treatment combinations were tested with three replicates each, and the Design Expert 13 software was used for statistical analysis to determine the effect of operating speed, hopper level, and plate hole shape on the dependent variable as putted on (Appendix B, Table B.1).

#### 2.5.1 Seed and fertilizer rate testing

In order to achieve the desired seed and fertilizer rate in the seeder the similar steps of Ref. [14] were follower.

1. Determine the width of planter (W)

(5) Width=Numberofrowxrowspacing

where, N = Number of furrow openers and S = Spacing between the furrow openers.

2. Calculate circumference of drive wheel

(6) L=πxd

where, d is the diameter of metering wheel.

3. Calculate area covered in one revolution,

(7) A=Lxw

(8) $$Seed\;rate=\frac{Weighted\;mass\;x10}{A_{t}},\text{kg}/\text{ha.}$$

The same as for fertilizer but time is 1 min.

#### 2.5.2 For compacter testing

For measuring the soil compaction as similar step of Ref. [11].

1) Test the moisture content of a prepared field with an HSM50 soil moisture meter.
2) Measure the cone index of the seedbed before compacting at 5 different positions of a field by using a cone penetrometer in the depth of 10 cm.
3) Weighting of 40 kg sand and filling in to the compactor cylinder and compacting the soil, test cone index at 5 different positions of the compacted field in the depth of 10 cm. Repeat for 50 kg and 60 kg.
4) Compare the dependent and independent variables (Appendix B Table B.2) and select the mass of sand that gives better compaction for the teff seed bed.

## 3 Result and discussion

Researchers built a machine to plant teff seeds and fertilizer. Testing the machine in a lab to see how well it worked at different speeds, hopper levels, and seed plate shapes [15]. They looked at how these settings affected the number of seeds planted, the amount of fertilizer applied, and whether seeds got stuck in the machine [16,17,18]. Design expert 13 software analyzed the data to determine optimal operating conditions by combining different factors [19,20,21,22]. Considering factors coded for plate shape (0-square, 1-cricular and 2- square one side inclined 45°), hopper level (0.25-quareter, 0.5 half and 1-full), and speed (2.11, 2.6 and 3.14 km/h) using one way and two way ANOVA to identify the most effective combination for planting efficiency. A total of 20 testing groups were set up in the simulation experiment. The response variables X1, and X2 were seed rate and fertilizer rate respectively. The effect of each variables are expressed by the quadratic regression equation since quadratic model suggested to fit summary. The experimental schemes and results are reported in Table B.3.

### 3.1 Response surface analysis of the seed rate (X1)

Table B. 4 shows ANOVA for response of seed rate to evaluate Quadratic model. It can be seen that A, B, C, AC, BC, A^2^, C^2^ have significant effect (P-values less than 0.0500) on fertilizer rate. The coefficients of the seed rate model responses using the factor codes as variables can be expressed as follows:

$$X1=+4.12-0.3750A+0.5130B+0.2010C\;-0.0425\text{AB}+0.1925\text{AC}+0.1500\text{BC}+0.1918A^{2}+0.0582B^{2}-0.4082C^{2}$$

**Fig. C.1:**
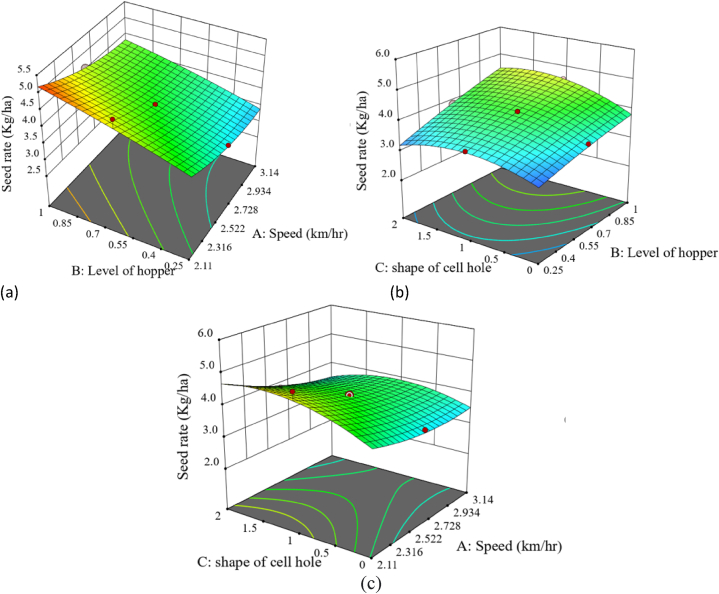
the response surfaces of the effects of interactive factors on the seed rate.

This research employed Response Surface Methodology to investigate the interactive effects of seeding speed, metering plate configuration, and hopper level on seed rate. Findings from Figure C.1a indicate a significant interaction between speed and hopper level on seed rate at the 5 % level of significance, as confirmed by two-way ANOVA (Appendix B, Table B.4). Increasing hopper level resulted in higher seed rates at a given speeds. Hopper level exerted a more pronounced influence on seed rate compared to speed, attributable to increased seed filling capacity within the metering plate holes. However, at optimal speed, the impact of hopper level diminished. Figure C.1b reveals a more substantial effect of metering plate configuration on seed rate than hopper level. When the plate hole shape changed from circular to square and square one side inclined 45° at a given level of hopper the seed rate starts to decrease due to the unfitting of the sphericity of teff with those shapes. Lastly, Figure C.1c demonstrates negligible variation in seed rate across different metering plate shapes and speeds. In the 20 run of variables the optimal value of seed rate is (4.8 kg/ha) which is get on circular shape and 2.6 km/h speed which is similar to the work of other [23,24].

### 3.2 Response surface analysis of the fertilizer rate (X2)

Table B.5 reports ANOVA for response of fertilizer rate to evaluate Quadratic model. P-values less than 0.0500 indicate model terms are significant. In this case A, B, AB, BC, A^2^, B^2^, C^2^ are significant model terms. The quadratic regression of the response of fertilizer rate in terms of coded factors as variables can be expressed as follows:

$$X2=+97.50-3.29A+1.96B+0.589C-0.9625AB+0.585AC-0.5075BC-1.47A^{2}+2.08\;B^{2}-3.63C^{2}$$

**Fig. C.2:**
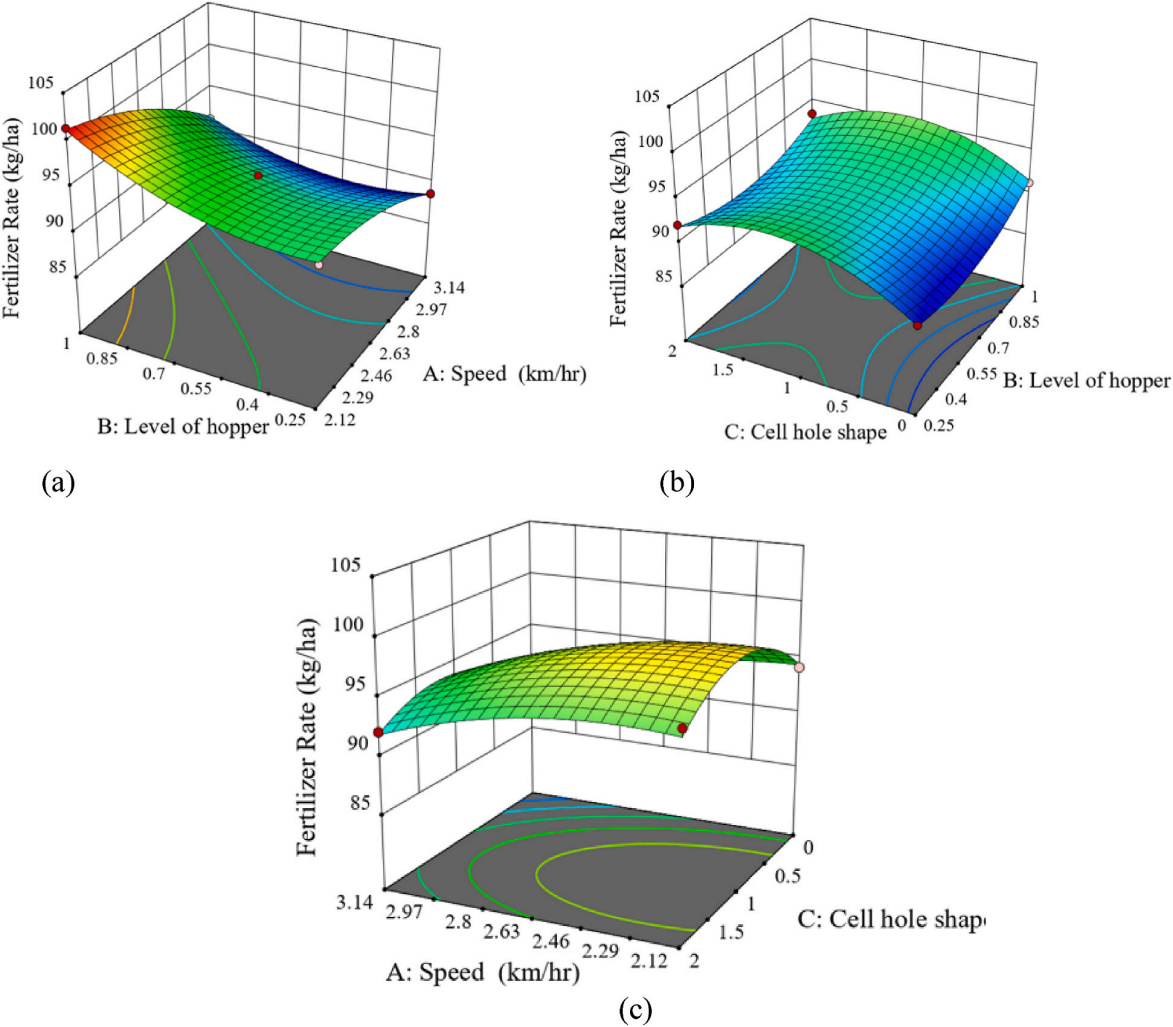
the response surfaces of the effects of interactive factors on the fertilizer rate.

Figure C.2a demonstrates a strong interaction between hopper level and operating speed on fertilizer application rate. When the operating speed increases at a given level of hopper, the fertilizer rate decreases. Conversely, at lower speeds, the increase in the level of hopper significantly boosts fertilizer rate. It is due to enough time to contact between fertilizer and metering plate holes, resulting in more fertilizer reaching the discharge tube without loss. However, Figure C.2bindicates that hopper level has a minimal influence on fertilizer rate for a specific plate hole configuration. Lower hopper levels and square plate holes correlate with reduced fertilizer rates, possibly due to the fertilizer's suboptimal sphericity for this configuration. Furthermore, Figure C.2c reveals a negligible impact of plate hole configuration on fertilizer rate when considering the interaction with speed. Variations in operating speed have limited effect on fertilizer rate at a given plate hole shape. During all trend of combination of variables the fertilizer rate varies from 97 to 100 kg/ha. But the optimal value of fertilizer rate by run of 20 factors is (100 kg/ha) get on 2.6 km/h at circular shape of cell hole that is approached to others output [25].

### 3.3 Compaction force

**Fig. C.3:**
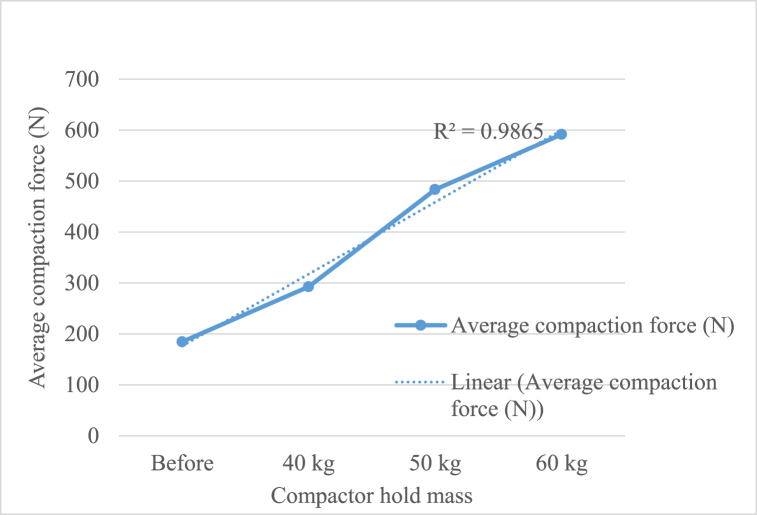
The relationship of compactor hold mass & average compaction force.

The relationship between compactor weight and compaction force was analyzed using data from Table B.6. A linear model was created in MS Excel 2016 to visualize this relationship (Figure C.3).

Comparing soil conditions before and after compaction revealed a significant improvement in cone index, with an accuracy of 98.65 %. This suggests that the uncompact soil provided poor seedbed conditions for small sized seed like teff, while compaction created a more favorable environment for seed germination and establishment. The compacted soil effectively held Teff seeds at the optimal planting depth, reducing lodging risk by improving root anchorage. As compactor weight increased, the average compaction force is also increase. The optimal weight was determined to be 50 kg, producing a force of 483.7 N. This force effectively compacted the soil to a depth of 20 mm over a width of 0.6 m, meeting the required compaction standards [11].

## 4 Conclusion

This research identified the limitations of traditional teff sowing methods, which are labor-intensive, wasteful, and hinder productivity. To address these challenges, develop a new animal-drawn machine that combines seedbed compaction with teff seed and fertilizer drilling. This machine features a unique vertical metering plate design as circular, square and square with 45° inclined in one side shapes and different hopper fill to control seed and fertilizer application. We conducted tests to determine the optimal operating conditions for the machine.

The results showed that a circular-shaped metering plate hole operating at 2.6 km per hour with almost all level of hopper delivered the desired teff seed rate (4.2 kg/ha) at 0.2865 unit of standard deviation and 7.2 % CV. Similarly, the same configuration achieved the target fertilizer application rate (96.2 kg/ha) for DAP particles. The circular shape of vertical metering plate, 2.6 km/h speed of operation and minimal impact of hopper fill level delivering the agronomical recommended amount of seed at a given rotation. Additionally, the compactor filled with 50 kg of sand at a specific moisture content proved most effective for compacting the teff seedbed.

Overall, this new machine offers several advantages over traditional methods. It reduces the need for animal trampling, allows for precise row sowing of seeds and fertilizer, and significantly reduces seed waste (by 81 %). Furthermore, by placing seeds at the proper depth, this method minimizes the risk of lodging (falling over) in teff plants due to a stronger root system.

## Data availability statement

The data that has been used is confidential and we will provide additional data upon request.

## CRediT authorship contribution statement

**Mulu Marie Takele:** Writing – review & editing, Writing – original draft, Software, Methodology, Investigation, Conceptualization. **Gedion Zelalem Dires:** Writing – review & editing, Writing – original draft, Validation, Software, Resources, Methodology, Investigation, Formal analysis, Data curation, Conceptualization. **Smegnew Moges Mintesnot:** Writing – review & editing, Visualization, Validation, Supervision, Software, Project administration, Investigation, Conceptualization. **Geta Kidanemariam Wagaw:** Validation, Supervision, Project administration, Investigation.

## Declaration of competing interest

The authors declare the following financial interests/personal relationships which may be considered as potential competing interests:

Mulu Marie Takele reports equipment, drugs, or supplies, statistical analysis, travel, and writing assistance were provided by Ethiopian Institute of Agricultural Research. Smegnew mogess mintesnot reports a relationship with 10.13039/501100004535Ethiopian Institute of Agricultural Research that includes: consulting or advisory and funding grants. Smegnew mogess mintesnot has patent pending to utility model. Geta Kidanemariam Gelaw was a reviewer of the ICAST and Heliyon journals.

## Figures and Tables

**Fig. A.2:**
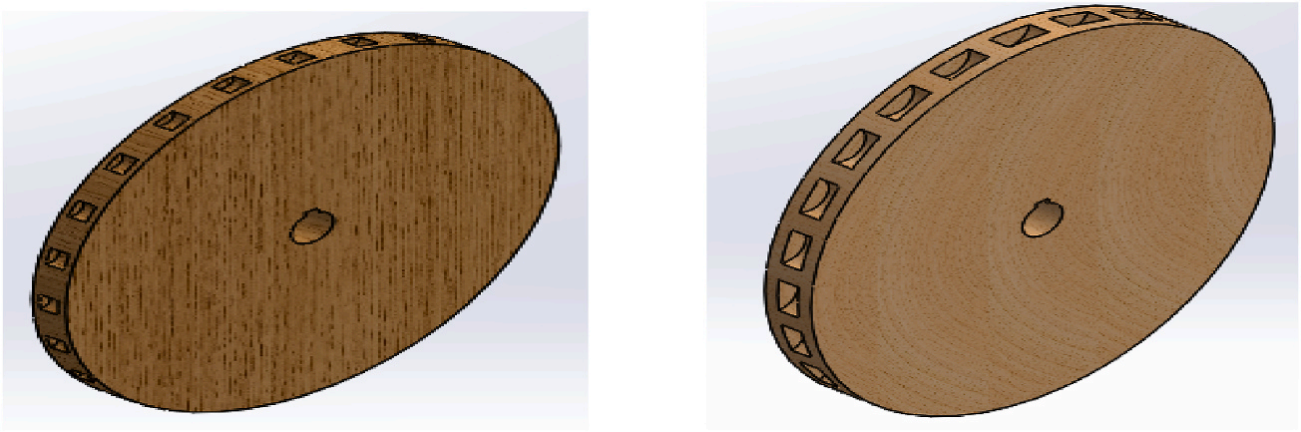
Seed and fertilizer metering plate.

**Table B.1:** Independent and dependent variables for metering plate

Independent variables	Treatment	Replication	Dependent variables
Metering plate hole shape	Circular	3	✓ Seed rate ✓ Fertilizer rate
	Square	3	
	45° inclined	3	
Forward Speed	N1	3	
	N2	3	
	N3	3	
Level of hopper	Full	3	
	Half	3	
	Quarter	3	

**Table B.2:** Independent and dependent variables for compactor testing

Independent variables	Treatment	Replication	Dependent variables
Mass of compactor cylinder hold	40 kg	5	Compaction force
	50 kg	5	
	60 kg	5	

**Table B.3:** The experimental design comprising three independent variables at three levels, and the simulation results of the responses

Run	A:Speed	B:hopper Level	C:Shape of plate hole	Response (X1)	A:Speed	B:hopper Level	C:Cell hole shape	Response (X2)
				Seed rate				Fertilizer rate
1	3.14	1	0	3.61	3.14	1	0	91.25
2	2.625	0.625	0	3.60	3.14	0.25	0	88.57
3	2.625	0.625	1	4.12	2.12	0.625	1	98.32
4	2.11	1	2	5.70	2.12	1	0	101.38
5	2.625	0.625	1	4.12	2.63	0.625	1	98.04
6	3.14	0.625	1	4.19	2.63	0.625	1	98.04
7	2.625	0.625	2	3.85	3.14	1	2	94.51
8	2.625	0.25	1	3.73	2.63	1	1	100.64
9	3.14	1	2	4.16	2.12	0.25	2	94.94
10	2.625	0.625	1	4.12	2.63	0.625	0	93.73
11	2.625	0.625	1	4.12	2.63	0.625	1	98.04
12	2.11	0.25	0	2.78	2.12	0.25	0	91.78
13	2.625	0.625	1	4.12	2.63	0.25	1	96.88
14	2.625	0.625	1	4.12	2.63	0.625	1	98.04
15	3.14	0.25	2	2.89	2.63	0.625	2	96.77
16	2.11	0.25	2	3.83	3.14	0.25	2	92.11
17	2.11	1	0	4.09	2.63	0.625	1	98.04
18	3.14	0.25	0	2.89	2.12	1	2	100.55
19	2.11	0.625	1	4.95	3.14	0.625	1	92.11
20	2.625	1	1	5.97	2.63	0.625	1	98.04

**Table B.4:** The ANOVA results of the quadratic model response and fit statistics to the seed rate

Source	Sum of Squares	df	Mean Square	F-value	p-value	
Model	5.57	9	0.6192	33.87	<0.0001	significant
A-speed	1.41	1	1.41	76.92	<0.0001	
B-level of hopper	2.63	1	2.63	143.95	<0.0001	
C-shape of cell hole	0.4040	1	0.4040	22.10	0.0008	
AB	0.0145	1	0.0145	0.7904	0.3949	
AC	0.2964	1	0.2964	16.22	0.0024	
BC	0.1800	1	0.1800	9.85	0.0105	
A^2^	0.1012	1	0.1012	5.53	0.0405	
B^2^	0.0093	1	0.0093	0.5092	0.4918	
C^2^	0.4582	1	0.4582	25.06	0.0005	
Residual	0.1828	10	0.0183			
Lack of Fit	0.1787	5	0.0357	43.77	0.0004	significant
Pure Error	0.0041	5	0.0008			

Std. Dev.	0.1352	R^2^	0.9682
Mean	3.98	Adjusted R^2^	0.9396
C.V. %	3.40	Predicted R^2^	0.7483
		Adeq Precision	25.7403

**Table B.5:** The ANOVA results of the quadratic model response and fit statistics to the fertilizer rate

Source	Sum of Squares	df	Mean Square	F-value	p-value	
Model	228.55	9	25.39	26.82	<0.0001	significant
A-Speed	108.04	1	108.04	114.11	<0.0001	
B-Level of hopper	38.42	1	38.42	40.57	<0.0001	
C-Cell hole shape	3.47	1	3.47	3.66	0.0846	
AB	7.41	1	7.41	7.83	0.0189	
AC	2.74	1	2.74	2.89	0.1199	
BC	2.06	1	2.06	2.18	0.1709	
A^2^	5.90	1	5.90	6.23	0.0316	
B^2^	11.90	1	11.90	12.57	0.0053	
C^2^	36.14	1	36.14	38.17	0.0001	
Residual	9.47	10	0.9468			
Lack of Fit	9.47	5	1.89			
Pure Error	0.0000	5	0.0000			

Std. Dev.	0.9731	R^2^	0.9602
Mean	95.99	Adjusted R^2^	0.9244
C.V. %	1.01	Predicted R^2^	0.7652
		Adeq Precision	18.9171

**Table B.6:** Compaction force and parameter

Parameter	Before compacting	After compacting in		
		40 kg	50 kg	60 kg
Average soil resistance (cone index) in KPa	558	944.3	1502.2	1845.5
Average compaction force (N)	180	304.1	483.7	594.3
